# Prolonged Continuous Theta Burst Stimulation of the Motor Cortex Modulates Cortical Excitability But not Pain Perception

**DOI:** 10.3389/fnsys.2020.00027

**Published:** 2020-05-29

**Authors:** Monika Klírová, Martin Hejzlar, Lenka Kostýlková, Pavel Mohr, Richard Rokyta, Tomáš Novák

**Affiliations:** ^1^Clinical Centre, National Institute of Mental Health, Klecany, Czechia; ^2^Department of Psychiatry, Third Faculty of Medicine, Charles University, Prague, Czechia; ^3^Department of Normal, Pathological and Clinical Physiology, Third Faculty of Medicine, Charles University, Prague, Czechia

**Keywords:** theta-burst stimulation, TBS, rTMS, cortical excitability, motor evoked potentials, pain, perception

## Abstract

Over the past decade, theta-burst stimulation (TBS) has become a focus of interest in neurostimulatory research. Compared to conventional repetitive transcranial magnetic stimulation (rTMS), TBS produces more robust changes in cortical excitability (CE). There is also some evidence of an analgesic effect of the method. Previously published studies have suggested that different TBS parameters elicit opposite effects of TBS on CE. While intermittent TBS (iTBS) facilitates CE, continuous TBS (cTBS) attenuates it. However, prolonged TBS (pTBS) with twice the number of stimuli produces the opposite effect. In a double-blind, placebo-controlled, cross-over study with healthy subjects (*n* = 24), we investigated the effects of various pTBS (cTBS, iTBS, and placebo TBS) over the right motor cortex on CE and pain perception. Changes in resting motor thresholds (RMTs) and absolute motor-evoked potential (MEP) amplitudes were assessed before and at two time-points (0–5 min; 40–45 min) after pTBS. Tactile and thermal pain thresholds were measured before and 5 min after application. Compared to the placebo, prolonged cTBS (pcTBS) transiently increased MEP amplitudes, while no significant changes were found after prolonged iTBS. However, the facilitation of CE after pcTBS did not induce a parallel analgesic effect. We confirmed that pcTBS with twice the duration converts the conventional inhibitory effect into a facilitatory one. Despite the short-term boost of CE following pcTBS, a corresponding analgesic effect was not demonstrated. Therefore, the results indicate a more complex regulation of pain, which cannot be explained entirely by the modulation of excitability.

## Introduction

Repetitive transcranial magnetic stimulation (rTMS) of the primary motor (M1) cortex modulates cortical excitability (CE) with subsequent neuroplastic changes observed in the stimulated area and its association areas, as well (Cárdenas-Morales et al., 2010). According to evidence-based guidelines, rTMS of the M1 cortex causes a significant clinical improvement in various neurological disorders, including neuropathic pain, fibromyalgia, post-acute stroke, and motor injury in Parkinson’s disease (Lefaucheur et al., 2020).

Recently, neurostimulatory research has become interested in theta-burst stimulation (TBS), a modification of high-frequency rTMS (HF-rTMS). There is evidence that TBS produces even more robust changes in CE than those observed in the conventional rTMS protocols (Gamboa et al., 2010; Suppa et al., 2016). Therefore, TBS offers the possibility to induce changes in CE with a more pronounced post-modulation effect in the regulation of corticospinal excitability and synaptic plasticity, with the potential to optimize clinical stimulation protocols (Gamboa et al., 2010).

TBS typically consists of bursts of three pulses at 30 Hz or 50 Hz, repeated five times per second with 600 pulses in total. There are two different paradigms: intermittent TBS (iTBS) and continuous TBS (cTBS). While iTBS facilitates CE (Huang et al., 2005; Di Lazzaro et al., 2008; Suppa et al., 2008, 2016), cTBS attenuates it (Di Lazzaro et al., 2005; Huang et al., 2005; Suppa et al., 2008; Goldsworthy et al., 2013; Wischnewski and Schutter, 2015). However, a prolonged form of cTBS (pcTBS) with twice the number of stimuli (1,200 pulses) produces a facilitatory effect similar to that of iTBS (Gamboa et al., 2010). By contrast, facilitatory iTBS (iTBS) is converted into inhibitory when applied for a period twice as long, which is called prolonged iTBS (piTBS; Gamboa et al., 2010). Moreover, CE can also be differently modulated for the TBS frequency used (30 Hz vs. 50 Hz), whereby its effect depends on time (minutes after TBS application) and interindividual differences between subjects (Chung et al., 2016).

It has been suggested that the analgesic effects of HF-rTMS of M1 stimulation result from the changes in pain modulation systems related to the long-term changes of neuronal excitability initiated by stimulation-induced changes in CE (Moisset et al., 2016), whereas the analgesic effect of TBS on the M1 cortex is explained by changes in excitability and ongoing activity in the connected areas that subsequently affect CE in M1 (Suppa et al., 2016).

Stimulation-induced pain relief is mainly attributed to the modulation of pain processing at the level of inhibition of the emotional response regions, such as the dorsolateral prefrontal cortex and the anterior cingulate cortex (ACC). Also, several other mechanisms that function through various neural pathways have been implicated, including the pain descending inhibitory system, whose activation at the brain stem level may lead to the inhibition of nociceptive transmission in the dorsal horn (Garcia-Larrea and Peyron, 2007; Leung et al., 2009).

An analgesic effect of HF-rTMS has been demonstrated in both experimental (Summers et al., 2004; Yoo et al., 2006; Nahmias et al., 2009; Borckardt et al., 2011) and clinical (Lefaucheur et al., 2001; Hirayama et al., 2006; Passard et al., 2007) pain studies. Furthermore, it has also been validated by several reviews and meta-analyses (Leung et al., 2009; Galhardoni et al., 2015; Goudra et al., 2017; Guo et al., 2017; Lefaucheur et al., 2020).

Similarly, TBS as a novel HF-rTMS approach has shown an analgesic effect in several inhibitory cTBS studies with healthy subjects (Poreisz et al., 2008; Csifcsak et al., 2009; Torta et al., 2013; Dowdle et al., 2019), while facilitatory iTBS studies have yielded predominantly negative results (Antal and Paulus, 2010; Borckardt et al., 2011; Houzé et al., 2013). Interestingly, in clinical trials with chronic pain patients, both cTBS and iTBS failed to affect the pain threshold and induce an analgesic effect (Lefaucheur et al., 2012; Gaertner et al., 2018). Only subjective transient pain relief has been observed in studies that used facilitatory iTBS as a priming protocol before HF-rTMS (Lefaucheur et al., 2012) or administered iTBS alone (Kohútová et al., 2017; Kim et al., 2020).

Regarding pTBS, only two studies with healthy subjects using pcTBS examined the analgesic effect, with both yielding positive results (Moisset et al., 2015; De Martino et al., 2019) despite different cortical areas were targeted (M1 and prefrontal cortex, respectively).

Evidence of a correlation between a stimulation-induced change in cortical CE of the M1 cortex and a change in the thermal pain threshold is mostly based on HF-rTMS trials in patients with pain (Lefaucheur et al., 2006; Mhalla et al., 2011). The absence of a post-stimulatory change in CE in the presence of an analgesic effect confirmed in several studies in healthy subjects is usually explained by the principle of homeostatic metaplasticity (Turrigiano, 2008). Healthy subjects without impaired homeostatic regulation do not display any significant post-stimulatory changes in CE (Moisset et al., 2016). Nevertheless, post-stimulatory changes in CE in healthy subjects have been repeatedly demonstrated in past experimental, non-pain studies, especially with (p)TBS (Huang et al., 2005; Di Lazzaro et al., 2008; Suppa et al., 2008, 2016; Gamboa et al., 2010; Goldsworthy et al., 2013; Wischnewski and Schutter, 2015).

It is also assumed that the analgesic effect of stimulatory protocols, particularly inhibitory cTBS in healthy subjects, is a consequence of cortical downregulation, whereas, in patients with chronic pain, the facilitatory HF-rTMS /iTBS leading to upregulation of CE is effective in pain relief (Antal and Paulus, 2010). These opposite results may be explained by the difference in the processing of acute provoked pain in healthy subjects and chronic pain symptoms, which are associated with maladaptive neuroplastic processes (Antal and Paulus, 2010). Interestingly, pcTBS studies have also shown that facilitatory pcTBS results in analgesic effects in healthy subjects (Moisset et al., 2015; De Martino et al., 2019). Overall, the exact mechanisms of action of rTMS-induced analgesic effects remain unclear and depend on many stimulation parameters.

Previously documented effects of pTBS prompt further investigation. Several studies reported an analgesic effect of inhibitory cTBS, as well as analgesia induced by facilitatory pcTBS, although it is not expected to be effective in relieving pain in healthy subjects. Presumably, an inhibitory piTBS protocol would induce analgesia in healthy subjects similar to other inhibitory protocols, including cTBS. So far, no studies in healthy subjects have tested whether inhibitory piTBS can reduce pain sensitivity.

Our primary objective was to test the effect of various pTBS protocols on CE of the M1 area. A secondary objective of the study was to investigate whether these protocols demonstrate an effect on the perception of pain. The effects of two different protocols of active pTBS (pcTBS, piTBS) were compared to placebo stimulation (plcTBS).

## Materials and Methods

### Subjects

Healthy volunteers (HV) of both sexes, aged between 18 and 45 years, free of pain during the past 6 months and without analgesic medication, were invited to participate. Exclusion criteria were current diagnosis or history of pain, organic brain disorder or injury, any other serious medical condition that may interfere with the rTMS/TBS administration (e.g., epilepsy, metallic plates in the head), pregnancy or breast-feeding, psychiatric disorder including history of a substance-induced disorder, except for nicotine addiction, and sensory or motor impairment that would preclude participation in the study. All of the participants signed an informed consent form, following the latest version of the Declaration of Helsinki, the study protocol was approved by the Independent Ethics Committee of the National Institute of Mental Health, Klecany.

### Study Protocol (Experimental Design)

**Figure 1:**
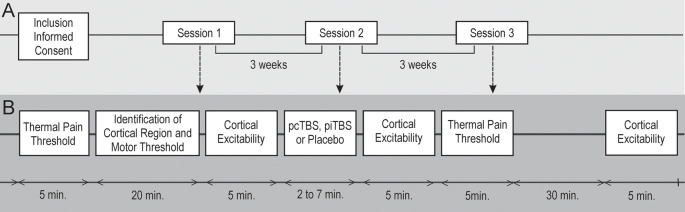
Schematic overview of the whole study. A double-blind, cross-over study. Three prolonged theta-burst stimulation (pTBS) sessions **(A)** were carried out in random order with a minimum 3-week wash-out period. Healthy subjects (*n* = 24) were randomly assigned to a sequence of three pTBS conditions **(B)** over the right M1 cortex: continuous pTBS (pcTBS); intermittent pTBS (piTBS); placebo pTBS (plcTBS). Cortical excitability (CE) changes in resting motor thresholds (RMTs) and absolute motor-evoked potential (MEP) amplitudes were assessed before and at two time-intervals (0–5 min; 40–45 min) after pTBS. Tactile and thermal pain thresholds quantitative sensory tests (QST) were measured before and 5 min after pTBS application.

The experiment was designed as a double-blind, placebo-controlled, cross-over study with three experimental phases, carried out in a random order, after a wash-out period of 3 weeks or longer (Figure 1A). Participants were randomly assigned to one of six different sequences of three TBS conditions (pcTBS, piTBS, or plcTBS), following William’s design. To assure an equal number of subjects in each sequence, we used non-stratified blocked randomization with a block size of six, computer-generated (www.randomization.com). Each experimental session was carried out at the same time of day, on the same weekday. Participants were asked to abstain from caffeine for at least 4 h before the TBS session and refrain from alcohol and any medication for 24 h before the TBS administration.

Both the study participants and the evaluating physicians were blind to the stimulation conditions and parameters. Quantitative Sensory Tests (QST), including measurement of tactile and thermal pain thresholds with subsequent measurement of resting motor thresholds (RMTs) and motor-evoked potential (MEP) amplitudes at the level of individual RMT, were performed before each pTBS session. During the pTBS sessions, the subjective acceptability of an individual stimulatory protocol was assessed. MEP amplitudes at the level of individual RMT with subsequent measurement of RMT after each pTBS session were evaluated immediately at the end of the pTBS application and then 40 min after the session. Tactile threshold and thermal/pain threshold at the end of pTBS application were measured 5 min after each stimulation session. Following the completion of the experiment, the subjects stayed in an idle state for the next 2 h. Subsequent stimulation sessions with a different stimulation protocol and an identical assessment schedule followed after 3, and 6 weeks, respectively (Figure 1B).

### pTBS Administration

TBS was administered using a MagPro R30 stimulator (Magventure^®^, Inc., Denmark) with a cool-B65 A/P figure-of-eight-shaped coil designed for double-blind research (the symmetrical design of the coil prevents the active vs. placebo side from being identified) with accessory two surface electrodes attached on the subject’s head for placebo stimulation. Surface electrodes used to stimulate skin sensation for plcTBS were also attached to the head during the active stimulation, to obtain identical settings. The coil was oriented tangentially to the scalp and horizontally in the anterior-posterior direction, which proved to be more effective in terms of pain relief than lateromedial positioning (André-Obadia et al., 2008; Andre-Obadia et al., 2018). TBS was administered over the contralateral motor cortex (right M1 area), specifically to the site corresponding to the somatotopic location of the left hand (thenar). The cortical area was located with a cool-B65 figure-of-eight-shaped coil by targeting with a single TMS pulse that induced a contralateral MEP of maximum amplitude in the left thenar (abductor pollicis brevis) while obtaining EMG responses with the accessory MEP monitor (EMG MagPro R30 equipment, Magventure^®^).

### Parameter Settings

pcTBS was applied with an intensity of 90% RMT, three pulses at 30 Hz in repetition after 200 ms continuously, 1,200 pulses per session in total, 1 min and 20 s duration;

piTBS was applied with an intensity of 90% RMT, three pulses at 30 Hz in repetition after 200 ms intermittently within the interval of 1,800 ms, with a train of 10 bursts and 8 s intertrain, 1,200 pulses per session in total, 6 min and 24 s duration;

plcTBS was applied with an A/P cool-B65 butterfly coil with an adjustable output for current stimulation of the subject’s skin, producing a surface current synchronous with magnetic stimulation pulses. During the placebo protocol, the coil with oppositely oriented and simultaneously shielded magnetic stimulation pulses were applied to the same location as during the active session. Placebo current stimulation, generating similar sensory stimuli as the active stimulation protocols, was administered *via* two surface electrodes placed on the subject’s right forehead and right temporal area.

### Assessments

Motor CE was examined by single-pulse TMS paradigms, which included the measurement of RMT and MEP amplitudes at rest (Lefaucheur et al., 2012). The analgesic effect of TBS was measured with QST (Rolke et al., 2006), evaluating changes of pain threshold, specifically the changes in the thermal pain threshold. The measurement of the threshold for tactile sensation by von Frey testing (Johansson et al., 1980) enabled undisturbed mechanical sensitivity to be confirmed and is also a part of the QST.

All of the subjects enrolled in the study underwent measurement of CE (RMT, MEP amplitude) with a MagProR30 device and MEP Monitor accessory. The measurement was taken in a quiescent state avoiding active muscle contraction before using TBS. CE was assessed at the site corresponding to the somatotopic location (left thenar) of the stimulated area of the M1 cortex and was examined before and after each pTBS application and 40 min after each pTBS application, according to the protocol adapted from Moisset (Moisset et al., 2015). RMT was assessed as the lowest intensity, expressed as the percentage of maximum stimulatory output needed to elicit five or more electromyographic responses (EMG MagPro R30 equipment) ≥50 μV within ten trials (Rossini et al., 1994). Individual values of MEP amplitudes within ten trials at the level of baseline individual RMT were measured and averaged (absolute MEP amplitude), and post-TBS MEP amplitudes were normalized by baseline average MEP amplitudes (normalized MEP amplitude).

All of the study subjects were also evaluated with QST that examined tactile and pain perception in the left (corresponding to the stimulated area of the M1 cortex) and right thenar eminences. They were assessed at the baseline and 5 min after each pTBS application (preceded by CE measurement). First, the tactile threshold was measured, then the thermal pain threshold was examined to eliminate the potential effect of the thermal generator on sensitivity disruption at the corresponding site. The tactile threshold was tested by the von Frey method (touch-test sensory evaluators, North Coast Medical). The thermal pain threshold was measured with a portable thermal stimulator of Algic stimuli (Yamamotová et al., 2017), a uniquely modified device that creates thermal stimulations with steadily increasing temperature (20–70°C), until a participant indicates, by pressing the left button of a computer mouse, their first perception of the relevant sensation and then the first perception of pain (Summers et al., 2004).

After each stimulation, the study subjects also self-evaluated tolerability and acceptability (pain and general discomfort) of the different stimulation protocols, with a 10-point subjective Visual Analog Scale (VAS; from 0 representing the absence of pain or discomfort to 10 being unacceptable pain; Khedr et al., 2005).

### Statistical Analyses

Subjective acceptability of TBS procedures assessed on a 10-point scale change across TBS conditions was compared by Friedman’s test with consequent Wilcoxon’s sign-ranked tests. The effect of TBS on CE parameters (absolute MEP amplitude and RMT) was calculated with repeated measures analysis of variance (RM ANOVA) including sequence as a between-subject factor, and treatment (piTBS, pcTBS, plcTBS) and time (baseline, immediately after, and after 40 mins) as within-subjects factors. For thermal thresholds, another within-subject factor, side, was added to the RM ANOVA model. Sphericity assumption was assessed by Mauchly’s test and if a violation was detected, then degrees of freedom were adjusted using Huynh–Feldt correction. If there was a significant treatment × time interaction, simple main effects (one-way model) were calculated for time and treatment, and paired *t*-tests with Bonferroni’s correction for multiple comparisons were consequently applied in the event of significant outcomes. For tactile threshold (ordinal scale) change comparisons, Friedmans ANOVA with Wilcoxons test was used. To assess inter-individual variability in MEP changes after TBS, the number of responders (>10% increase from the baseline) and inverse responders (>10% decrease from the baseline) was compared between conditions using Cochrane’s Q test. Pearson’s correlation coefficient was used to analyze relationships between the changes in thermal thresholds and normalized CE parameters (after/baseline). The thermal threshold changes between responders and non-responders, as well as between females and males under the respective TBS conditions, were compared using Welsh’s *t*-test.

A sample size of 24 subjects should enable detecting effect size ($\eta_{\text{p}}^{2}$) of 0.14 or larger in the RM ANOVA model for within-subjects simple main effects (time and treatment), at a given alpha of 0.05, power (1-beta) of 0.90, and estimated correlation of repeated measurements of 0.3, if present.

All of the statistical analyses were performed using the STATISTICA 12 software (StatSoft, Inc., 2008). All of the tests were two-sided and *p* < 0.05 was regarded as being statistically significant.

## Results

Twenty-four HV (mean age 30.4 ± 3.3 years, 11 females) were enrolled in the study and randomized to treatment sequences. Twenty-three subjects completed all three treatment conditions and were entered into further analyses. One subject dropped out of the study after pcTBS (female; sequence pcTBS → piTBS → plcTBS) due to procedural intolerance. The mean baseline RMT for the whole sample was 48.7 ± 8.6% (range 35–66%) of the maximum device output.

Both pTBS sessions were less tolerated than plcTBS; piTBS was less tolerated than pcTBS [VAS: piTBS: median 3 (IQR 1–6); pcTBS 1 (0–3), plcTBS 0 (0–0); Friedman’s ANOVA *χ^2^* = 22.1, *p* < 0.001; *post hoc*: piTBS vs. pcTBS; *z* = 3.52, *p* < 0.001]. Four subjects considered the pain felt during piTBS as being almost unbearable (VAS ≥7). No other serious side effects were reported.

**Figure 2:**
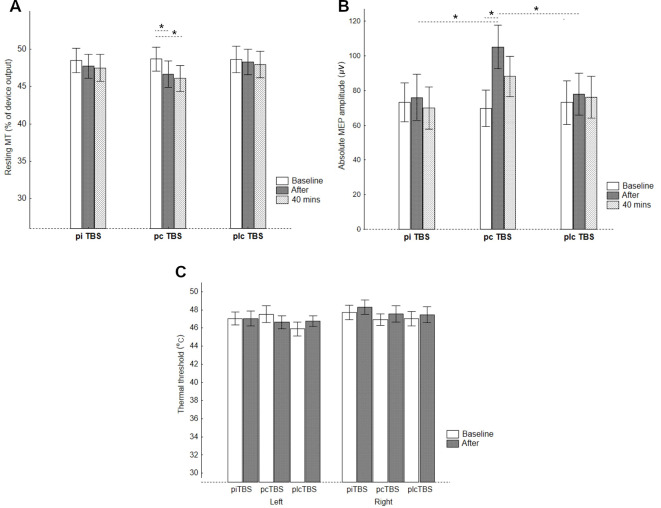
CE represented by **(A)** RMTs, **(B)** absolute MEP amplitudes, and pain perception represented by **(C)** thermal thresholds for the thenar of the left and right thenar eminences after prolonged TBS (pTBS). pcTBS: continuous pTBS; piTBS: intermittent pTBS; plcTBS: placebo TBS. The data are presented as mean ± SE. *p < 0.05 after Bonferroni’s test.

When analyzing the RMT change, RMANOVA revealed a significant treatment × time interaction (*F*_(4,68)_ = 2.76, *p* = 0.03, $\eta_{\text{p}}^{2}$ = 0.14), and main effect of time (*F*_(1.7,29.3)_ = 8.94, *p* = 0.001, $\eta_{\text{p}}^{2}$ = 0.34), but a non-significant effect of treatment (*F*_(1.9,31.7)_ = 1.14, *p* = 0.33, $\eta_{\text{p}}^{2}$ = 0.06). Neither effect of sequence (*F*_(5,17)_ = 1.62, *p* = 0.22) nor sequence × treatment interaction (*F*_(9.1,31.7)_ = 1.13, *p* = 0.37) was significant. Subsequent analysis indicated the only significant simple main effect of time for pcTBS (*F* = 9.03, *p* = 0.001) and Bonferroni’s *post hoc* test showed a significant decrease in RMT for both post-TBS measurements (immediately: −1.96, 95%CI −0.37 to −3.55, *p* = 0.01; 40 min: −2.61, −1.02 to −4.20, *p* < 0.001) compared to the baseline. The simple main effects of treatment, however, only indicated a trend towards a significant difference between conditions after 40 min (immediately: *F* = 2.08, *p* = 0.14; 40 min: *F* = 3.17, *p* = 0.052; Table 1, Figure 2A).

**Table 1:** Cortical excitability (absolute and normalized MEP amplitude, and RMT), thermal thresholds and tactile thresholds after prolonged theta burst stimulation (pTBS).

		Baseline	After	40 min after
**Absolute MEP (μV)**	**piTBS**	72.9 ± 49.3	75.7 ± 57.7	70.2 ± 53.0
	**pcTBS**	69.0 ± 47.1	100.7 ± 57.9*^#^^†^	84.2 ± 54.1
	**plcTBS**	73.0 ± 53.4	78.1 ± 52.1	75.8 ± 53.4
**Normalized MEP**^1^				
	**piTBS**		1.05 ± 0.32	0.98 ± 0.33
	**pcTBS**		1.61 ± 0.71^#^^†^	1.29 ± 0.53^†^
	**plcTBS**		1.11 ± 0.25	1.07 ± 0.34
**RMT (% of device output)**	**piTBS**	49.0 ± 8.5	48.4 ± 8.7	48.2 ± 9.1
	**pcTBS**	49.2 ± 8.8	47.4 ± 9.6*	46.7 ± 9.1*
	**plcTBS**	49.3 ± 8.7	49.0 ± 8.6	48.7 ± 8.9
**Thermal threshold (°C)**^2^			
	**piTBS**	47.1 ± 3.3	47.0 ± 3.9	
	**pcTBS**	47.5 ± 4.3	46.5 ± 3.4
	**plcTBS**	46.0 ± 3.6	46.7 ± 2.8	
**Tactile threshold (g)**^2^	**piTBS**	1.73 ± 0.22	1.74 ± 0.22
	**pcTBS**	1.77 ± 0.28	1.71 ± 0.20	
	**plcTBS**	1.73 ± 0.22	1.71 ± 0.17	

Data are presented as mean ± SD. piTBS: prolonged intermittent TBS; pcTBS: prolonged continuous TBS; plcTBS: placebo TBS. *p < 0.05 vs. baseline; ^#^*p* < 0.05 vs. sham; ^†^*p* < 0.05 vs. piTBS after Bonferroni’s test. ^1^Normalized MEP—post-TBS MEP amplitudes normalized by baseline average MEP amplitudes. ^2^Data for thermal and tactile thresholds are presented as an average from both sides.

Analysis of absolute values of MEP amplitude at the level of the individual baseline RMT found significant treatment × time interaction (*F*_(4,68)_ = 5.58, *p* < 0.001, $\eta_{\text{p}}^{2}$ = 0.25), and effect of time (*F*_(2,34)_ = 7.83, *p* = 0.002, $\eta_{\text{p}}^{2}$ = 0.32), but no significant effect of treatment (*F*_(1.8,30.4)_ = 1.38, *p* = 0.38, $\eta_{\text{p}}^{2}$ = 0.05). The effects of sequence (*F*_(5,17)_ = 0.38, *p* = 0.85), and sequence × treatment interaction (*F*_(8.9,30.4)_ = 0.71, *p* = 0.69) were non-significant. Furthermore, the simple main effects of time revealed a significant increase in absolute MEP amplitude after pcTBS (*F* = 11.73, *p* < 0.001), but not after either piTBS (*F* = 0.71. *p* = 0.50) or plcTBS (*F* = 0.66, *p* = 0.52). *Post hoc* comparisons found a significantly increased absolute value of MEP amplitude after pcTBS (34.6, 95%CI 16.8–52.3, *p* < 0.001) but not after 40 min (17.3, −0.4 to 35.1, *p* = 0.058). The simple main effect of treatment was significant for measurement immediately after (*F* = 4.48, *p* = 0.016), but not after 40 min (*F* = 1.03, *p* = 0.36). The increase in MEP amplitudes after pcTBS was higher compared to both plcTBS (25.4, 1.8–49.0, *p* = 0.036) and piTBS (28.0, 4.4–51.7, *p* = 0.02; Table 1, Figure 2B).

Also, 6, 19, and 10 subjects were classified as responders (>10% increase of MEP after compared to before) to piTBS, pcTBS, and plcTBS, respectively (Cochran’s Q test: *Q* = 13.3, *p* = 0.001). A decrease in MEP amplitude >10% (inverse responders) was found in 4, 1, and 5 and no change in 13, 3, and 8 subjects after piTBS, pcTBS, and the placebo, respectively.

In contrast to the CE parameters, no significant effect of TBS on thermal threshold was found (main effect of time (*F*_(1,17)_ = 0.77, *p* = 0.39, $\eta_{\text{p}}^{2}$ = 0.04), treatment (*F*_(2,34)_ = 0.64, *p* = 0.53, $\eta_{\text{p}}^{2}$ = 0.04), side (*F*_(1,17)_ = 2.62, *p* = 0.12, $\eta_{\text{p}}^{2}$ = 0.13), and treatment × time interaction (*F*_(2,34)_ = 0.71, *p* = 0.50, $\eta_{\text{p}}^{2}$ = 0.04, observed power of 0.13; Table 1, Figure 2C). Similarly, we failed to find any significant effect on tactile perception (Friedmans ANOVA: *χ^2^* = 1.47, *df* = 5, *p* = 0.9; Table 1).

Concerning the relationship between CE parameters and thermal thresholds, only a tendency toward a significant correlation between normalized MEP amplitude and thermal threshold change after piTBS was found (*r* = −0.41, *p* = 0.06). Otherwise, no significant correlation was revealed between changes in CE parameters and thermal threshold across the conditions (pcTBS *r* = 0.18, *p* = 0.40; plcTBS *r* = 0.07, *p* = 0.77). Furthermore, there was no difference in thermal threshold changes between responders and non-responders (piTBS: *t* = 1.26, *p* = 0.23; pcTBS: *t* = 0.83, *p* = 0.46; plcTBS: *t* = 0.81, *p* = 0.43) or between genders (piTBS: *t* = −0.66, *p* = 0.52; pcTBS: *t* = −0.03, *p* = 0.98, *t* = −0.10, *p* = 0.92).

## Discussion

Our findings confirmed the effect of pcTBS on the facilitation of CE, demonstrated by the increase of MEP amplitudes and the decrease of RMT. Compared to other stimulation protocols used in our study (piTBS and plcTBS), we found the pcTBS paradigm to be the only effective intervention. Moreover, the continuous form of pTBS was also the best tolerated active stimulation protocol.

The results are fully congruent with the hypothesis that by doubling the stimulation duration of the prolonged form of cTBS, conventional inhibitory cTBS converts into facilitatory cTBS. The results are also in agreement with the results of a previously published study testing the effect of pTBS on CE (Gamboa et al., 2010). The use of a different frequency in our study (30 Hz instead of 50 Hz) did not change the outcome. On the other hand, piTBS did not affect any of the tested variables; therefore, we could not confirm the earlier observation that the piTBS would induce the change, i.e., immediate decrease CE in a group of healthy subjects (Gamboa et al., 2010). This could be attributed to the fact that, unlike Gamboa’s study, we measured CE on RMT, but not on an active motor threshold (AMT).

Another possible explanation for CE facilitation after pcTBS, but not CE suppression after piTBS, maybe the fact that we were biased in detecting the potentially piTBS-induced CE suppression by measuring MEPs below the testable range. In an earlier study with non-prolonged TBS, the authors found that cTBS-induced MEP inhibition is observed by measuring at higher stimulus intensities (150% RMT), while lower stimulus intensities (110% RMT) are optimal for detecting iTBS-induced MEP facilitation (Goldsworthy et al., 2016). Based on their results, it may be assumed that potential piTBS-induced MEP inhibition will be observed when probed with a stimulus intensity much higher than at an RMT level.

However, our findings do not support the results reported in a previously published pTBS study with healthy subjects (Moisset et al., 2015). The authors failed to detect the change of CE after pcTBS but observed its analgesic effect. None of the pTBS protocols used in our study induced analgesia; specifically we did not observe any effect of pTBS on the thermal/pain threshold changes. Therefore, our results do not allow us to establish a relationship between the change of CE and analgesia induced by intracortical modulation with pTBS in healthy subjects. Nevertheless, different techniques used to measure pain threshold should be mentioned as a possible explanation for dissimilar findings compared to previous pain-related pTBS studies (Moisset et al., 2015; De Martino et al., 2019). Also, we cannot exclude type II error risk if true differences in thermal thresholds are moderate-sized.

There is a lack of rTMS/TBS studies in healthy subjects that examined the correlation between the change of CE and thermal/pain threshold changes. The correlation has been reported mostly in clinical rTMS trials of patients with pain (Lefaucheur et al., 2006; Mhalla et al., 2011). We assume that the effect of rTMS/TBS adjusts impaired thermal detection through the activation of systems that modulate the long-term change in neuronal excitability in patients with pain (Moisset et al., 2016). However, if the thermal sensation of healthy individuals is not disturbed, the correlation between the change of CE and the change of thermal threshold may not occur.

Recent contradictory data from TBS studies suggest that apart from the total number of pulses administered during the stimulation session, the varying effect (both on CE and pain threshold) may also be explained by the impact of other stimulation parameters (Goldsworthy et al., 2012). According to a TBS meta-analysis of non-prolonged cTBS (pcTBS) studies (using 600 pulses per session) that compared the effect of different stimulation frequencies (30 Hz vs. 50 Hz) on MEP suppression, the changes in the 30 Hz TBS subgroup were more persistent (Chung et al., 2016).

Besides, we cannot ignore the fact that other interindividual differences, such as the influence of BDNF polymorphism on the change of the amplitude of MEP after rTMS/TBS (Jacobs et al., 2014; Chung et al., 2016) may also play an important role in the effect of TBS (Jannati et al., 2017). Therefore, investigation of repeated measures within an individual may produce more reliable results than interindividual group comparisons (Suppa et al., 2016).

There are several possible sources of heterogeneity among experimentally-induced pain rTMS studies to be considered. It is supposed that various rTMS paradigms affect A-delta-fiber and C-fiber-mediated induced pain in different ways (Leo and Latif, 2007). Also, stimulation-induced changes in pain perception in some rTMS (Summers et al., 2004; Lefaucheur et al., 2010; de Andrade et al., 2011) and TBS (Csifcsak et al., 2009; Torta et al., 2013; Moisset et al., 2015; De Martino et al., 2019) studies may be explained by the various measurement methods used. Laser and cold stimulation are standard methods in measuring pain perception. While acute laser-induced pain is mediated by activation of the A-delta-fiber pathway, cold-induced pain is mediated by a combination of the A-delta-fiber and C-fiber pathways (Leo and Latif, 2007). Moreover, the thermal stimulator, with the gradually induced heat pain used in our study, presumably employs a mechanism of activation mediated by both the A-delta-fiber and the C-fiber pathways (Kostek et al., 2016). Therefore, it can be assumed that our measurement of the pain threshold adequately reflects previous (p)TBS studies (Moisset et al., 2015; De Martino et al., 2019).

The main limitation of our study is that, unlike other pTBS studies (Moisset et al., 2015; De Martino et al., 2019), we did not include non-pcTBS or conventional HF-rTMS as a positive control to verify the analgesic effect previously demonstrated by HF-rTMS/cTBS studies (Summers et al., 2004; Yoo et al., 2006; Nahmias et al., 2009; Borckardt et al., 2011). However, for testing stimulation-induced changes of CE, HF-rTMS would not be appropriate as a positive control since previous studies showed that the after-effects of rTMS-induced change in CE were highly variable and inhomogeneous among individuals (Maeda et al., 2000; Pell et al., 2011).

Similarly to previous pTBS studies (Moisset et al., 2015; De Martino et al., 2019), we determined CE based on RMT, while AMT would be a better criterion for adjusting the strength of TBS application (Huang et al., 2005; Gamboa et al., 2010; Lefaucheur et al., 2012). This is another limitation of our study, since AMT more reliably sets a defined state, while RMT may vary greatly according to the subject’s attention in motor preparation and spatial attention (Mars et al., 2007).

We should point out that the determination of CE based on RMT, levels of MEP amplitude, is a rather indirect measure. Currently, TMS-evoked EEG responses are preferred as a more direct measure of CE since the RMT and MEP amplitudes may differ for other reasons than changes in CE. Moreover, the after-effects of rTMS and TBS are highly variable between individuals (Hamada et al., 2013).

Another limitation of our study is the absence of a neuronavigation system to target the area for stimulation according to the structural/functional imaging examination (specifically MRI, fMRI). In general, neuronavigated rTMS/TBS allows a better reproducibility and accuracy regarding the definition of the stimulation and potentially greater efficacy of the method (Moisset et al., 2016). It should be noted that the accuracy of the functional targeting with the coil focused on the specific M1 area (corresponding to the somatotopic location of the left hand), that enables electromyographic responses of the corresponding muscles to be immediately detected, is similar to focusing with neuronavigation (Herwig et al., 2002).

Current knowledge suggests that pain relief induced by HF-rTMS and TBS is mainly attributed to induced changes in neuronal excitability in the areas associated with emotional pain processing, particularly the ACC modulation. Therefore, it would be of interest to test the effect of (p)TBS by deep stimulation (e.g., with H-coil) targeted directly to the rostral part of the ACC and to investigate its potential analgesic effect.

In conclusion, following previously published research documenting pcTBS-induced changes in CE of the M1 cortex, our results suggest that pcTBS represents a well-tolerated, non-invasive method that increases CE. We confirmed that a prolonged form of cTBS with twice the duration converts the conventional inhibitory effect into a facilitatory one. In contrast to previous studies, we failed to demonstrate the analgesic effect of pcTBS. Further studies are needed to clarify these contradictory findings. Our results indicate more complex regulation of pain that cannot be explained entirely by the modulation of M1 excitability. Future studies, especially with TMS-evoked EEG responses, should examine pTBS-induced changes in neuronal excitability in associated brain areas that are responsible for pain processing.

## Data Availability Statement

The datasets generated for this study are available on request to the corresponding author.

## Ethics Statement

The studies involving human participants were reviewed and approved by Independent Ethics Committee (IEC) of the National Institute of Mental Health, Klecany, Czechia. The patients/participants provided their written informed consent to participate in this study.

## Author Contributions

MK contributed to the study conception and design analysis, data collection, interpretation of results, and drafting and revision of the article. MH contributed to data acquisition and assembly and interpretation of the data. LK contributed to the acquisition, assembly, and analysis of the data. PM contributed to the conception and design, and drafting and revision of the article. RR contributed to conception and design, interpretation of data, and revision of the article. TN contributed to conception and design analysis of data, interpretation of data, and drafting and revision of the article.

## Conflict of Interest

The authors declare that the research was conducted in the absence of any commercial or financial relationships that could be construed as a potential conflict of interest.
